# Molecular Basis for Immunity Protein Recognition of a Type VII Secretion System Exported Antibacterial Toxin

**DOI:** 10.1016/j.jmb.2018.08.027

**Published:** 2018-10-19

**Authors:** Timothy A. Klein, Manuel Pazos, Michael G. Surette, Waldemar Vollmer, John C. Whitney

**Affiliations:** 1 Michael DeGroote Institute for Infectious Disease Research, McMaster University, Hamilton, Canada L8S 4K1; 2 Department of Biochemistry and Biomedical Sciences, McMaster University, Hamilton, Canada L8S 4L8; 3 Centre for Bacterial Cell Biology, Institute for Cell and Molecular Biosciences, Newcastle University, Newcastle upon Tyne, NE2 4HH, United Kingdom; 4 Department of Medicine, Farncombe Family Digestive Health Research Institute, McMaster University, Hamilton, Canada L8S 4K1

**Keywords:** T7SS, type VII secretion system, VSV-G, vesicular stomatitis virus glycoprotein G, type VII secretion system, antibacterial toxin, toxin-immunity pair, interbacterial competition, protein-protein interactions

## Abstract

Gram-positive bacteria deploy the type VII secretion system (T7SS) to facilitate interactions between eukaryotic and prokaryotic cells. In recent work, we identified the TelC protein from *Streptococcus intermedius* as a T7SS-exported lipid II phosphatase that mediates interbacterial competition. TelC exerts toxicity in the inner wall zone of Gram-positive bacteria; however, intercellular intoxication of sister cells does not occur because they express the TipC immunity protein. In the present study, we sought to characterize the molecular basis of self-protection by TipC. Using sub-cellular localization and protease protection assays, we show that TipC is a membrane protein with an N-terminal transmembrane segment and a C-terminal TelC-inhibitory domain that protrudes into the inner wall zone. The 1.9-Å X-ray crystal structure of a non-protective TipC paralogue reveals that the soluble domain of TipC proteins adopts a crescent-shaped fold that is composed of three α-helices and a seven-stranded β-sheet. Subsequent homology-guided mutagenesis demonstrates that a concave surface formed by the predicted β-sheet of TipC is required for both its interaction with TelC and its TelC-inhibitory activity. *S. intermedius* cells lacking the *tipC* gene are susceptible to growth inhibition by TelC delivered between cells; however, we find that the growth of this strain is unaffected by endogenous or overexpressed TelC, although the toxin accumulates in culture supernatants. Together, these data indicate that the TelC-inhibitory activity of TipC is only required for intercellularly transferred TelC and that the T7SS apparatus transports TelC across the cell envelope in a single step, bypassing the cellular compartment in which it exerts toxicity en route.

## Highlights

• Antibacterial TelC toxin is neutralized in the inner wall zone by membrane-anchored TipC immunity protein.
• TipC is a crescent-shaped protein that interacts with TelC via its concave surface.
• TelC and TipC are physically separated by the plasma membrane in TelC-producing cells.
• The type VII secretion system prevents TelC access to the inner wall zone in TelC-producing bacteria.

## Graphical abstract

**Figure:**
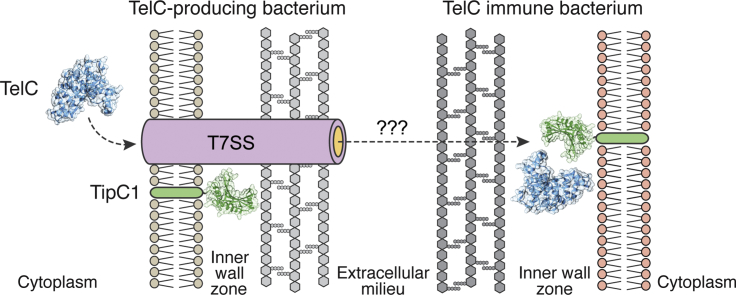

## Introduction

Bacteria employ a variety of mechanisms to transport macromolecules across membranes. One of the ways this process is accomplished in Gram-positive bacteria is through a multi-subunit membrane protein complex known as the type VII secretion system (T7SS) [1]. T7SSs are best studied in the phylum Actinobacteria where they have been shown to facilitate the transport of molecules involved in a wide array of biological processes. For example, the mycobacterial ESX-1, ESX-3 and ESX-4 T7SSs have been implicated in the lysis of host cell membranes, siderophore-mediated iron uptake and conjugal DNA transfer, respectively [2], [3], [4]. The T7SS has also been characterized in the low G + C Gram-positive phylum Firmicutes, which possesses an evolutionarily distant subfamily of this pathway referred to as T7SSb [1]. The T7SSb apparatus comprises fewer protein subunits than Actinobacterial T7SSs and functions to mediate protein export from the cell [5]. Among T7SSb-containing bacteria, the *ess* locus of *Staphylococcus aureus* is the most extensively characterized. This system exports four small non-enzymatic proteins of unknown function named EsxA, EsxB, EsxC and EsxD, which belong to the WXG100 family of T7SS effectors [6], [7]. In addition, the large nuclease toxin EsaD is exported in a T7SS-dependent manner, and phenotypic characterization of *S. aureus* strains lacking the *esaD* gene indicates that this toxin contributes to both the bacteria and host cell-targeting capabilities of this pathway [8], [9].

Recently, we demonstrated that *Streptococcus intermedius* uses its T7SS for antagonistic bacterial cell–cell interactions, further substantiating the bacteria-targeting capability of the T7SSb pathway [10]. *S. intermedius* is a commensal bacterium found within the densely populated microbial flora of the human oral cavity and is also an opportunistic pathogen [11]. In addition to the WXG100 protein EsxA, we demonstrated that the T7SS of *S. intermedius* exports three effector proteins named TelA, TelB and TelC [10]. The Tel proteins belong to the large and broadly distributed LXG family of polymorphic toxins and the discovery of these effectors provided the first experimental evidence that this family of toxins transits the T7SS [12]. While the mode of action of TelA is unknown, biochemical characterization of TelB demonstrated that it exerts toxicity by degrading the electron carrying dinucleotide NAD^+^, whereas TelC functions as a phosphatase that cleaves peptidoglycan precursor lipid II.

Concomitant with our discovery of the Tel proteins was the finding that each of these effectors is encoded in close proximity to a gene that encodes a toxin-specific immunity protein [10]. For example, TelA and TelB are toxic in the bacterial cytoplasm and their cognate immunity proteins, TipA and TipB, confer immunity to their respective toxins when expressed in this cellular compartment. Furthermore, TelB-expressing strains of *S. intermedius* exhibit a fitness advantage when grown in co-culture with *S. intermedius* strains lacking TipB [10]. These observations suggest that cytoplasmic immunity proteins protect bacteria from both self-produced toxins and toxins delivered by sister cells via the T7SS. Since TipA and TipB were not identified as substrates of the T7SS, cytoplasmic TelA–TipA and TelB–TipB complexes are presumably dissociated prior to toxin export as has been observed for other interbacterial polymorphic toxin delivery systems [13].

TelC is distinct from characterized Gram-positive antibacterial toxins because it acts in the inner wall zone when delivered into target bacteria by the T7SS [10]. Consequently, the TelC-specific immunity protein TipC1 may differ from TipA and TipB in that it likely localizes to this cellular compartment to enable it to confer immunity to TelC. If this prediction is true, TipC1 would be physically separated from its cognate effector in TelC-producing cells by the plasma membrane. T7SS effector translocation across the plasma membrane is catalyzed by the FtsK/SpoIIIE-like motor ATPase EssC [14]; however, it is not known if the T7SS apparatus additionally facilitates effector transport across the thick Gram-positive cell wall. Thus, it is unclear if TipC1 is required for protection from self-produced TelC or if it is only needed to confer immunity to intercellularly delivered TelC.

In the present work, we sought to uncover the site of action and mode of TelC inhibition by the TipC immunity protein. To this end, we used subcellular localization and protease accessibility assays to show that TipC is a membrane protein with an extracellular TelC-inhibitory domain. We then determined the structure of a non-protective TipC paralogue, which allowed for homology modeling of TipC. Mutagenesis analysis informed by this structural model suggests that TipC inhibits TelC toxicity via a concave surface formed by a seven-stranded β-sheet. Finally, mutational inactivation of *tipC* does not render *S. intermedius* cells susceptible to self-produced TelC, although the toxin is exported from the cell via the T7SS. Taken together, these data point to a model in which TipC is required for protection from competitor delivered but not self-produced TelC toxin.

## Results

### TipC localizes to the plasma membrane via an N-terminal transmembrane domain

**Fig. 1:**
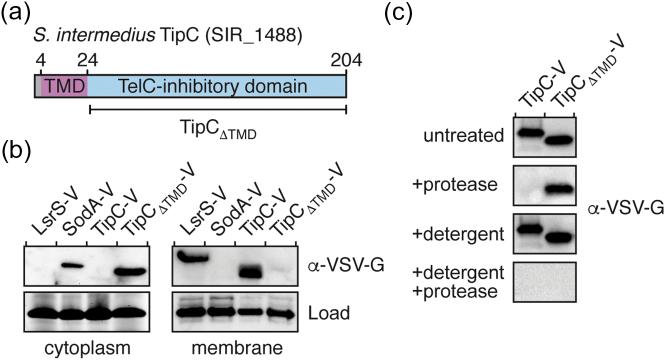
TipC is a surface exposed membrane protein. (a) Domain organization of TipC from *S. intermedius* B196. The boundaries for the TelC-inhibitory domain (TipC_ΔTMD_) and the predicted transmembrane domain (TMD) are indicated. (b) TipC1 is anchored to the plasma membrane via its N-terminal TMD. Western blot analysis of the cytoplasmic and membrane fractions of *S. intermedius* B196 strains expressing the indicated VSV-G epitope (V) tagged proteins. SodA-V and LsrS-V are cytoplasmic and membrane protein controls, respectively. Stain-free detection (Bio-Rad) was used to ensure equal loading between samples. (c) The TelC-inhibitory domain of TipC is surface exposed. Western blot analysis of *S. intermedius* B196 spheroplasts expressing TipC-V or TipC_ΔTMD_-V. Spheroplasts were treated with Proteinase K (protease), Triton X-100 (detergent) or both and compared to an untreated control.

We previously showed that the soluble region of TipC is sufficient to inhibit the toxic lipid II phosphatase activity of TelC *in vitro* [10]. In these biochemical assays, a truncated form of TipC that excluded its hydrophobic N-terminus was employed in order to reduce TipC aggregation in aqueous buffer, and consequently, a functional role for this region of the protein was not determined (Fig. 1a). Lipid II exists in both the inner and outer leaflet of the plasma membrane; however, for reasons that are unclear, TelC only exerts toxicity when targeted to the inner wall zone [10]. We hypothesized that the ability of TipC to effectively neutralize a toxin that acts in the inner wall zone on a membrane-embedded substrate arises because the protein itself also localizes to the plasma membrane. To test this, we performed subcellular fractionation experiments on *S. intermedius* B196 (Si^B196^) cells expressing vesicular stomatitis virus glycoprotein G (VSV-G) epitope-tagged TipC (TipC-V). The characterized streptococcal proteins manganese-dependent superoxide dismutase (SodA) and *l*antibiotic *S*mb *r*eceptor-like function in *s*treptococci (LsrS) were used as cytoplasmic and membrane protein fractionation controls, respectively [15], [16]. Consistent with our hypothesis, we found that TipC localizes to the membrane fraction (Fig. 1b). Furthermore, this localization was dependent on the hydrophobic N-terminus of TipC because a truncated form of TipC lacking this region of the protein (TipC_ΔTMD_) was found exclusively in the cytosol.

We next examined the orientation of the TelC-inhibitory domain of TipC in the plasma membrane. Our previous finding that TipC abrogates toxicity caused by Sec translocon-targeted TelC suggested that this domain exists in the inner wall zone [10]. To test this prediction, we performed protease accessibility assays on spheroplasts generated via lysozyme digestion of Si^B196^ cells expressing TipC or TipC_ΔTMD_ [17]. As shown in Fig. 1c, only full-length TipC was readily degraded by the added protease. In contrast, cytoplasmic TipC_ΔTMD_ was susceptible to proteolysis only after spheroplast rupture by detergent. Together, these data are consistent with the prediction that TipC is a membrane protein with a TelC-inhibitory soluble domain that protrudes into the inner wall zone.

### *telC*–*tipC* operons harbor multiple *tipC* paralogous genes

**Fig. 2:**
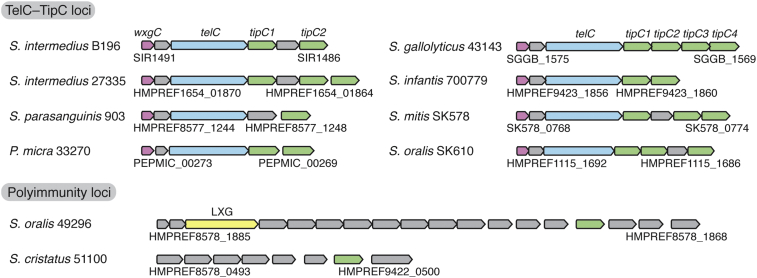
*telC* gene clusters possess multiple *tipC* orthologous genes. Genomic context of *tipC* orthologous genes from representative Firmicute species. Genes are colored according to homology and by known or predicted function of the encoded protein (TelC-interacting chaperones, purple; TelC toxins, blue; TipC immunity proteins, green; uncharacterized LXG toxin, yellow; other, gray).

Having established a functional role for the N-terminal TMD of TipC, we next sought to identify the region of its C-terminal domain responsible for its TelC-inhibitory activity. Similar to the T7SS-exported Tel proteins, antibacterial toxins delivered by other pathways involved in interbacterial antagonism possess cognate immunity proteins that protect toxin-producing bacteria from the activity of their own toxins and/or toxins delivered intercellularly by sister cells [18]. These immunity proteins are highly specific toward their cognate toxin as pairs of homologous immunity proteins with greater than 50% identity between them have been shown to have opposing abilities to neutralize a given toxin [19]. We sought to exploit this observation to identify amino acid residues critical for the TelC-inhibitory activity of TipC by locating variable positions between TipC homologous sequences. BLASTp analysis of the NCBI non-redundant sequence database identified 286 TipC homologous proteins whose distribution is restricted to species belonging to the order *Lactobacillales*. Examination of the genomic context of *tipC* ORFs revealed that the vast majority of these genes exist in operons with similar synteny to that of Si^B196^ (Fig. 2). In addition, we noted two examples of *tipC* genes found in gene clusters that may represent heterogeneous arrays of immunity genes as defined by Zhang and colleagues [12]. Of particular utility to this work, we also found that the majority of *tipC*-containing bacteria possess multiple *tipC* paralogous genes. Si^B196^ possesses one such *tipC* paralogous gene (SIR_1486), which encodes a protein with 58% identity to TipC. To disambiguate these two proteins, we henceforth refer to TipC (SIR_1488) as TipC1 and the protein encoded by SIR_1486 as TipC2.

### TipC2 does not protect against TelC-mediated toxicity

**Fig. 3:**
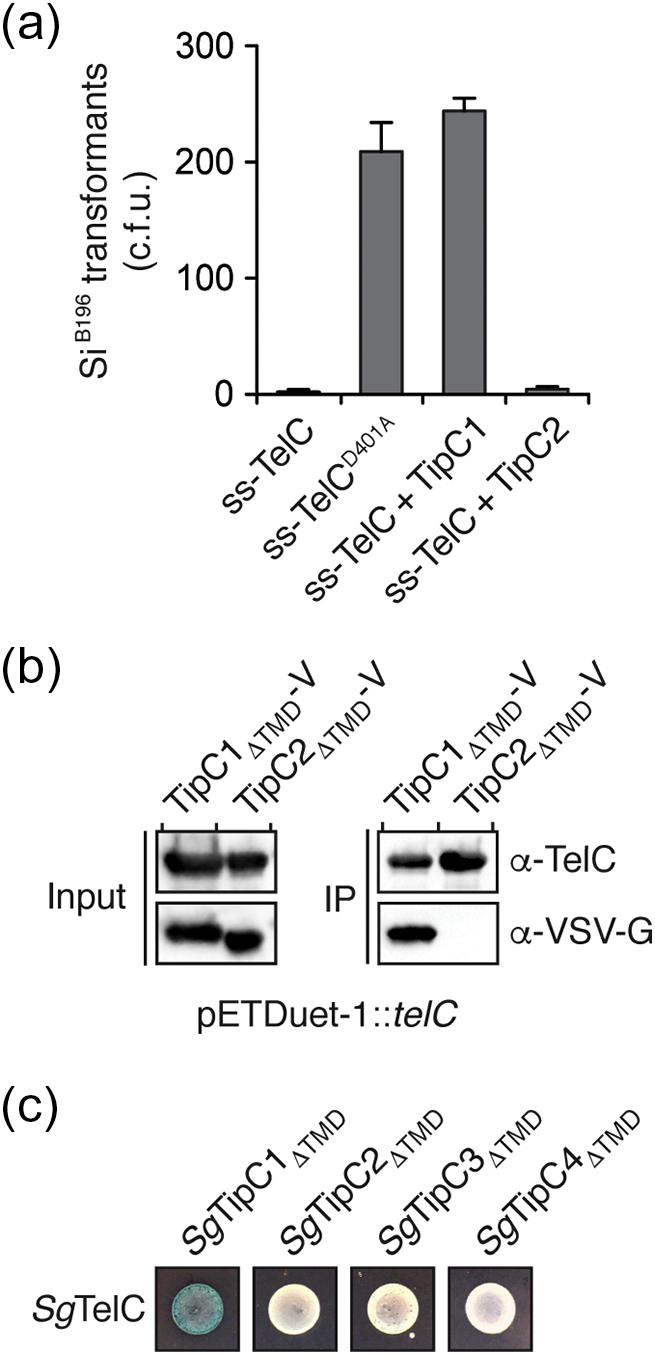
TipC2 does interact with TelC or confer immunity to TelC-mediated toxicity. (a) Number of *S. intermedius* B196 colonies after transformation with equimolar amounts of a plasmid constitutively expressing the indicated proteins. TelC fused to a Sec signal peptide (ss-TelC) and an inactive variant thereof (ss-TelC^D401A^) serve as positive and negative controls, respectively. Details on the construction of these plasmids have been described previously [10]. Error bars represent ± SD (*n* = 3). (b) TipC2_ΔTMD_ does not interact with TelC. VSV-G epitope-tagged TipC1_ΔTMD_ (TipC1_ΔTMD_-V) and TipC2_ΔTMD_ (TipC2_ΔTMD_-V) were co-expressed with his_6_-tagged TelC and assessed for co-purification by Western blot analysis. (c) Only the *telC* adjacent *tipC* gene of *S. gallolyticus* ATCC 43143 encodes a protein (*Sg*TipC1) capable of interacting with the TelC orthologous protein (*Sg*TelC) from this organism. Bacterial two-hybrid analysis of *Sg*TelC and each of the four TipC orthologous proteins from *S. gallolyticus* ATCC 43143. *Sg*TelC was fused to the T25 fragment of adenylate cyclase and co-expressed with each TipC orthologous protein fused to the T18 fragment. Blue color indicates a protein–protein interaction. A schematic of the *S. gallolyticus* ATCC 43143 *telC-tipC* gene cluster can be found in Fig. 2.

Given the high degree of homology between TipC1 and TipC2, and the observation that slight divergence in immunity protein sequence is sufficient to abrogate toxin-inhibitory activity [19], it seemed reasonable that this protein could guide our identification of TipC1 residues that mediate TelC inhibition. Toward this end, we first tested whether TipC2 could protect Si^B196^ from TelC-based toxicity. In contrast to cells expressing TipC1, we found that TipC2 expression could not prevent toxicity caused by constitutive expression of the TelC toxin domain (TelC_tox_) targeted to the inner wall zone of Si^B196^ (ss-TelC_tox_) (Fig. 3a). To rule out that the failure of TipC2 to inhibit TelC activity is a result of inherent instability of the protein, we next performed nickel affinity co-purification experiments using his_6_-tagged TelC_tox_ co-expressed with VSV-G epitope-tagged TipC1 or TipC2. To simplify the purification process, we used TipC1_ΔTMD_ and a similarly truncated TipC2 variant that also lacks its N-terminal transmembrane domain (TipC2_ΔTMD_) because we previously showed that this region of TipC1 is not required for its ability to inhibit the toxic lipid II phosphatase activity of TelC *in vitro* [10]. As shown in Fig. 3b, these experiments demonstrated that although both TipC1_ΔTMD_ and TipC2_ΔTMD_ accumulate to substantial levels in cells, only TipC1_ΔTMD_ is capable of interacting with TelC. We further expanded this line of inquiry to an organism possessing more than two *tipC* paralogous genes. *Streptococcus gallolyticus* ATCC 43143 contains four adjacently encoded TipC proteins (*Sg*TipC1–4). Using bacterial two-hybrid analysis, we found that only *Sg*TipC1_ΔTMD_ is capable of interacting with *S. gallolyticus* TelC (*Sg*TelC) (Fig. 3c). Together, these results indicate that in two different bacteria, TipC1 proteins, but not downstream encoded paralogous TipCs, possess the molecular determinants for cognate TelC inhibition.

### X-ray crystal structure of TipC2_ΔTMD_

Our finding that TipC1_ΔTMD_, but not TipC2_ΔTMD_, interacts with and confers immunity to TelC substantially reduces the number of potential residues that could be involved in TelC inhibition. However, structure prediction algorithms were unable to generate a high-confidence model of TipC1_ΔTMD_ that would allow us to predict which candidate residues are surface exposed and thus be more likely to interact with and inhibit TelC. Crystallization efforts failed to yield diffraction quality crystals of TipC1_ΔTMD_ or TelC–TipC1_ΔTMD_ complex; however, TipC2_ΔTMD_ readily crystallized in the space group *C*2, and we were able to determine its X-ray crystal structure to 1.8-Å resolution using selenium-incorporated protein and the selenium single wavelength anomalous dispersion technique [20]. The resulting electron density maps allowed for complete model building of TipC2_ΔTMD_ (residues 23–203) and a vector-encoded proline residue derived from the linker region connecting a his_6_-tag to the N-terminus of TipC2_ΔTMD_. The final model was refined to an *R*_work_/*R*_free_ of 17.0% and 19.4%, respectively (Table 1).

**Table 1:** X-ray data collection and refinement statistics

	TipC2_ΔTMD_ (selenomethionine)
Data collection	
Beamline	ALS 5.0.2
Wavelength (Å)	0.979
Space group	*C*2
Cell dimensions	
*a*, *b*, *c* (Å)	159.7, 54.5, 104.4
*α*, *β*, *γ* (°)	90.0, 108.0, 90.0
Resolution (Å)	33.60–1.75 (1.78–1.75)^a^
Total no. of reflections	85,866
*R*_merge_ (%)^b^	4.8 (140.8)^a^
*I*/σ*I*	21.1 (1.3)^a^
Completeness (%)	99.2 (98.5)^a^
Redundancy	7.3 (6.7)^a^

Refinement	
*R*_work_/*R*_free_ (%)^c^	17.0/19.4
No. atoms	
Protein	4489
Water	489
Average *B*-factors (Å^2^)	
Protein	37.5
Water	32.3
Rms deviations	
Bond lengths (Å)	0.014
Bond angles (°)	1.221
Ramachandran plot (%)^d^	
Total favored	96.2
Total allowed	100.0
Coordinate error (Å)^e^	0.18

a Values in parentheses correspond to the highest-resolution shell.

b *R*_merge_ = ΣΣ |* I*(*k*) − <* I* >|/Σ*I*(*k*), where *I*(*k*) and <* I* > represent the diffraction intensity values of the individual measurements and the corresponding mean values. The summation is over all unique measurements.

c *R*_work_ = Σ ||* F*_obs_ | − *k* |* F*_calc_ ||/|* F*_obs_ |, where *F*_obs_ and *F*_calc_ are the observed and calculated structure factors, respectively. *R*_free_ is the sum extended over a subset of reflections excluded from all stages of the refinement.

d As calculated using MOLPROBITY [47].

e Maximum-likelihood based coordinate error, as determined by PHENIX [48].

**Fig. 4:**
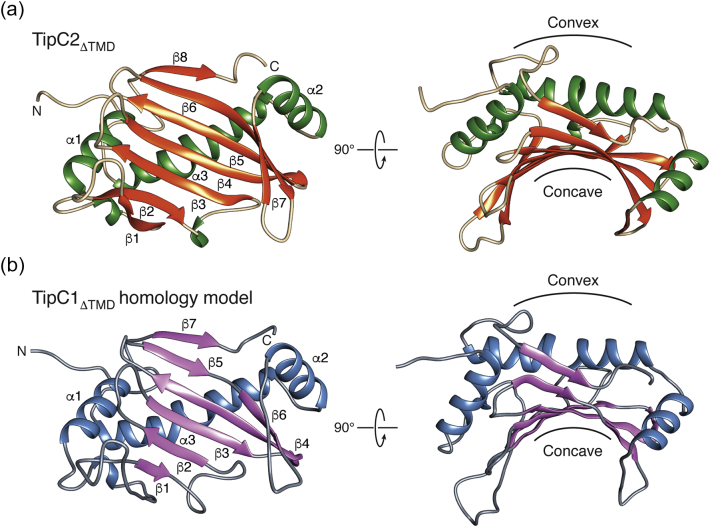
X-ray crystal structure of TipC2_ΔTMD_ and homology model of TipC1_ΔTMD_. (a) Overall structure of TipC2_ΔTMD_ shown as a ribbon representation and viewed from two orthogonal angles. (b) I-Tasser-generated homology model of TipC1_ΔTMD_ shown as a ribbon representation and viewed from two orthogonal angles. Secondary structure elements and the concave and convex surfaces of both proteins are indicated.

TipC2_ΔTMD_ adopts a mixed α/β fold consisting of three α-helices and seven β-strands that fold together to give the protein a distinct crescent-shaped appearance (Fig. 4a). This shape is characterized by a concave surface formed by a seven-stranded β-sheet and a convex surface generated by the positions of three α-helices. Using the DALI webserver to compare our structure with all deposited structures in the PDB, we determined that TipC2_ΔTMD_ does not bear strong resemblance to proteins of known structure [21]. Many of the top scoring proteins from this analysis were outer-membrane proteins from Gram-negative bacteria whose β-strands loosely resemble the β-sheet of TipC2_ΔTMD_. For example, the amyloid secretion protein FapF from *Pseudomonas* sp. UK4 and the oligogalacturonate-specific porin KdgM from *Dickeya dadantii* superimpose with TipC2_ΔTMD_ with Cα RMSDs of 4.4 and 3.9 Å, respectively, over 91 equivalent Cα positions. Also identified in this analysis was the polo box 1 (PB-1) domain of ZYG-1 Plk4 kinase from *Caenorhabditis* *elegans* (Cα RMSDs of 2.8 Å over 82 equivalent Cα positions). Although also functionally unrelated to TipC2, this crescent-shaped domain mediates a protein–protein interaction with the centriole duplication protein SPD-2 via its concave surface, suggesting that the equivalent surface on TipC1 may interact with TelC [22].

**Fig. 5:**
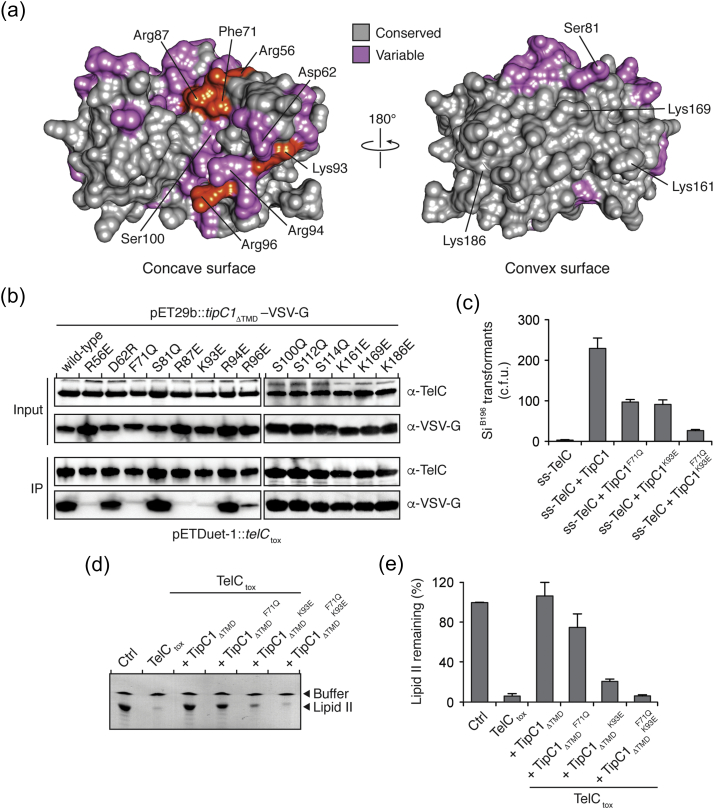
A concave surface of TipC1 mediates interaction with TelC. (a) Surface representation of a TipC1_ΔTMD_ homology model showing the concave and convex surfaces of the protein. Amino acid residues that are conserved (gray) or variable (pink) between TipC1_ΔTMD_ and TipC2_ΔTMD_ are depicted. Variable amino acids critical for interaction with TelC (red, defined in B) are labeled. (b) R56E, F71Q, R87E, K93E and R96E variants of TipC1_ΔTMD_ do not interact with TelC. VSV-G epitope-tagged wild-type TipC1_ΔTMD_ and the indicated TipC1_ΔTMD_ site-specific variants were co-expressed with his_6_-tagged TelC and assessed for co-purification by Western blot analysis. (c) Number of *S. intermedius* B196 colonies after transformation with equimolar amounts of a plasmid constitutively expressing the indicated proteins. Plasmids expressing ss-TelC and ss-TelC + TipC1 serve as positive and negative controls, respectively. Error bars represent ± SD (*n* = 3). (d) Thin-layer chromatography analysis of reaction products from incubation of synthetic Lys-type lipid II with buffer (Ctrl), TelC_tox_, TelC_tox_ and TipC1_ΔTMD_ or TelC_tox_ and the indicated TipC1_ΔTMD_ site-specific variants. (e) Densitometric quantification of (d). Error bars indicate ± SD (n = 3).

Because TipC2 does not protect cells from TelC-mediated toxicity, we next employed the I-Tasser structure threading server to generate a homology model of TipC1_ΔTMD_ (Fig. 4b) [23]. The resulting TipC1_ΔTMD_ model (residues 23–204) had a template modeling score of 0.65, indicating that the probability that our TipC1_ΔTMD_ model has the same overall topology and fold as TipC2_ΔTMD_ is greater than 95% [24], [25]. In addition, circular dichroism spectroscopy demonstrates that TipC1_ΔTMD_ and TipC2_ΔTMD_ have very similar secondary structure composition (Fig. S1). We next mapped the amino acids that vary between TipC1 and TipC2 onto the surface of the TipC1_ΔTMD_ model, restricting our selection to amino acid R-groups with differing polarity (Fig. 5a). This analysis revealed that the majority of conserved residues are found on the convex surface, while the variable residues were predominantly found on the concave surface. Taken together with our finding that TipC2_ΔTMD_ does not interact with TelC_tox_, these findings support the idea that the concave surface of TipC1 facilitates its interaction with TelC.

### The predicted concave surface of TipC1 harbors the molecular determinants for TelC binding

To dissect the interaction between TipC1_ΔTMD_ and TelC, we next performed homology model-guided mutagenesis on TipC1_ΔTMD_. A structure of a T7SS effector–immunity pair has not yet been determined; however, a number of co-crystal structures exist of effector–immunity complexes from Gram-negative polymorphic toxin systems [26], [27]. These structures show that the buried surface area between effectors and their cognate immunity proteins is substantial, typically exceeding 1000 Å^2^, and thus, these interactions may be difficult to disrupt by a conservative mutagenesis approach. Therefore, we mutated surface-exposed hydrophobic and small hydrophilic residues to the large, hydrophilic amino acid glutamine whereas charged amino acids were substituted with a residue of opposite charge. Each site-specific TipC1_ΔTMD_ variant was co-expressed with his_6_-tagged TelC_tox_, and binding was assessed via pull-down analysis. Importantly, all TipC1_ΔTMD_ variants tested expressed to comparable levels in *Escherichia coli*, indicating that these amino acid substitutions did not adversely impact the stability of the protein (Fig. 5b). In line with our structural analyses, we found that site-specific substitution of residues on the convex surface of TipC1_ΔTMD_ had no effect on the ability of the protein to interact with TelC_tox_. In contrast, mutation of arginine 56 (R56E), phenylalanine 71 (F71Q), arginine 87 (R87E), lysine 93 (K93E) and arginine 96 (R96E), all of which lie on the TipC1_ΔTMD_ concave surface, substantially reduced TelC_tox_ binding (Fig. 5a and b).

We next selected two of the identified TipC1_ΔTMD_ point mutants defective in TelC_tox_ binding, F71Q and K93E, and tested if these variants could rescue Si^B196^ cells from TelC-based toxicity. Individually, we found that these TipC variants exhibited a partial reduction in their ability to protect cells from the toxic activity of TelC while a TipC1 variant bearing both of these amino acid substitutions displayed a substantially greater reduction in TelC-neutralizing capability (Fig. 5c). Consistent with these findings, we found that only the TipC1_ΔTMD_ double mutant lacked no inhibitory activity toward the lipid II phosphatase activity of TelC_tox_ (Fig. 5D and E). This defect in TelC inhibition by the TipC1_ΔTMD_ double mutant is not due to misfolding of the protein as its circular dichroism spectrum was indistinguishable from wild-type TipC1_ΔTMD_ (Fig. S2). Together, these data indicate that the concave surface of TipC1 is required for direct inhibition of the toxic lipid II phosphatase activity of TelC.

### TelC bypasses the inner wall zone via the T7SS in TelC-producing cells

**Fig. 6:**
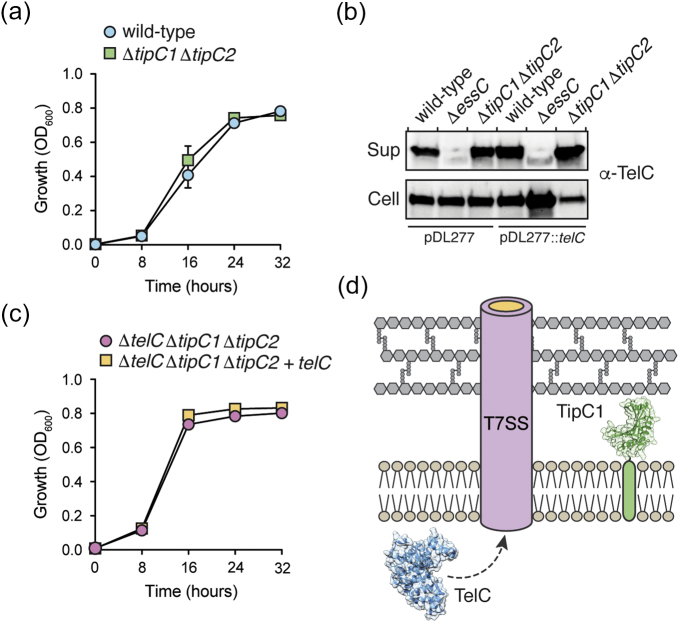
TelC does not access the inner wall zone as it transits the T7SS. (a) Mutational inactivation of *tipC* genes does not affect the growth of *S. intermedius* B196. Growth of the indicated *S. intermedius* B196 strains grown in liquid media. Error bars indicate ± SD (*n* = 3). (b) TelC expressed from its native locus or from a multi-copy plasmid accumulates in culture supernatants. Western blot analysis of TelC levels in supernatant (sup) or cell fractions of the indicated *S. intermedius* B196 strains. (c) Plasmid-borne expression of TelC in strains lacking *tipC* genes does not affect the growth of *S. intermedius* B196 strains grown in liquid media. Error bars indicate ± SD (*n* = 3). (d) Model depicting the T7SS-dependent export of TelC across the Gram-positive cell envelope in a single step.

Having established that TipC1 is a membrane protein with a soluble TelC-inhibitory domain that exists in the inner wall zone, we next wanted to exploit the unique site of action of TelC to gain insight into the export mechanism of the T7SS. Our prior finding that TelC is toxic to both *S. aureus* and Si^B196^ cells when artificially targeted to the Sec translocon but not when milligram quantities of purified, active TelC toxin are added to susceptible cells suggests that the Gram-positive cell wall prevents the diffusion of TelC between the extracellular milieu and the inner wall zone [10]. Taking these observations into consideration, we posited that the T7SS apparatus likely facilitates the export of effector proteins across the entire Gram-positive cell envelope in a single step. In this model, deletion of *tipC1* would be expected to have no detrimental effect on Si^B196^ growth in liquid media because TelC and TipC1 would be physically separated by the plasma membrane in toxin-producing cells and the T7SS would allow TelC to bypass the inner wall zone during export. Importantly, T7SS-dependent intercellular intoxication would not occur because this requires growth on a solid surface [10]. In contrast, if the T7SS only functions to export TelC from the cytoplasm to the inner wall zone, a *tipC1*-deficient strain would likely be susceptible to intoxication by self-produced TelC. To distinguish between these two possibilities, we generated a Si^B196^ strain lacking *tipC* genes and assessed whether this strain is susceptible to TelC-mediated toxicity by comparing its growth rate in liquid monoculture to that of its parent strain (Fig. 6a). Under these conditions, the immunity-deficient strain showed no significant growth impairment, although substantial amounts of the TelC toxin could be detected in culture supernatants (Fig. 6b). To rule out the possibility that endogenous levels of TelC are insufficient to observe intoxication by self-produced toxin, we also employed the plasmid-based expression system used for our Sec translocon-targeting TelC toxicity assays to express TelC in our immunity-deficient strain. Despite elevated levels of TelC accumulation in culture supernatants, this strain also exhibited no measurable growth defect in monoculture compared to immunity-expressing strains (Fig. 6b and c). When contrasted with our previous observation that TelC is toxic when targeted to the inner wall zone via a sec leader peptide [10], these data suggest that the T7SS apparatus forms a continuous channel that facilitates TelC export from the cytoplasm into the extracellular milieu in a single step (Fig. 6d).

## Discussion

This study describes the first biochemical characterization of a T7SS immunity protein. We have shown that TipC1 is a membrane protein with a soluble domain that localizes to the inner wall zone and is responsible for its TelC-inhibitory activity. Furthermore, using structural and informatic approaches, we identified a concave surface on TipC1 that mediates its direct interaction with TelC. By showing the dispensability of TipC1 in TelC-producing cells, we also provide evidence that T7SS effectors bypass the inner wall zone as they transit the secretory apparatus.

TipC1 is distinct from the other identified T7SS immunity proteins TipA, TipB and EsaG in that it neutralizes a toxin that acts from outside the cell. In Gram-negative bacteria, the antibacterial type VI secretion system (T6SS) has been shown to deliver toxins into the periplasm that similarly disrupt cell surface structures [28]. For example, the T6SS-delivered toxin Tse1 is a peptidoglycan hydrolase that, like TelC, possesses a cognate immunity determinant [29]. This immunity protein, named Tsi1, is a soluble periplasmic protein that inhibits Tse1 despite not being anchored to the cellular structure that it protects, presumably because the confines of the Gram-negative periplasm allow Tsi1 to accumulate to levels that confer resistance to Tse1-mediated toxicity [30]. Our finding that TipC1 is anchored to the plasma membrane not only increases the proximity of its TelC-inhibitory domain to the lipid II substrate of TelC but also prevents its diffusion into the extracellular milieu through the estimated 50-kDa molecular weight cutoff pores of the peptidoglycan layer [31]. Although peptidoglycan hydrolyzing toxins with cognate immunity proteins have yet to be identified in Gram-positive bacteria, should these toxins exist, the diffusion of their associated immunity proteins away from the cell could similarly be prevented via covalent tethering to the cell wall via an LPXTG sorting motif [32].

Like TelC, the Colicin M family of proteins includes antibacterial toxins with lipid II phosphatase activity [33]. Colicins differ from T7SS-exported toxins in that they act between closely related Gram-negative bacteria and they do not require a specialized secretion system for delivery; however, they are similar in that they possess cognate immunity proteins that confer resistance to toxin activity [34]. The structure of a colicin M immunity protein (Cmi) from *E. coli* has been solved in both monomeric and domain-swapped dimeric states [35], [36]. The Cmi dimer is approximately the same molecular weight as TipC; however, it does not bear any significant structural similarity. Furthermore, its overall shape is tetragonal, in contrast to the crescent-shaped appearance of TipC. The weak interaction between Colicin M and Cmi *in vitro* has made mapping their interaction interface challenging, and thus, it is unclear if the residues responsible for this interaction cluster to a discrete area of Cmi in a manner that is analogous to what we have shown here for TipC1. A lipid II phosphatase–immunity protein co-crystal structure is needed to provide further mechanistic insight into how this family of enzymes is inactivated by proteinaceous inhibitors.

We exploited inability of the TipC2 protein to inhibit TelC-mediated toxicity to identify TipC1 amino acids critical for its function. However, the observation that many *telC*-containing bacteria possess additional *tipC* genes whose protein products do not interact with the TelC protein of the same organism raises the question of what the function of these genes is. One intriguing possibility is that these additional genes confer immunity to TelC toxins produced by other bacterial species. If this is indeed the case, then these bacteria would be resistant not only to TelC delivered by sister cells but also from divergent TelC toxins delivered by other species of bacteria occupying the same niche. Lending further support to this hypothesis, we identified several bacteria that possess “orphan” *tipC* genes, which presumably exist to provide protection from intercellularly delivered TelC toxins.

The dispensability of *tipC* immunity genes in TelC-producing strains coupled with our observation that TelC targeted to the Sec translocon is toxic but TelC targeted to the T7SS is not, suggests that the T7SSb secretion apparatus exports its substrates across not only the plasma membrane but also the peptidoglycan layer. One way this might be accomplished is by a continuous proteinaceous channel formed by the structural components of the T7SS apparatus. To date, the best-characterized T7SS structural subunit is the EssC ATPase, which exports proteins across the plasma membrane via a mechanism that requires homo-multimerization [14]. However, the other structural components of the T7SSb pathway, such as EsaA, EssA and EssB, are less well characterized, and it remains to be determined if the complex formed by these proteins forms a channel that penetrates the peptidoglycan sacculus [37]. Recently, a “needle-like” structure was shown to be formed by the EspC protein of the mycobacterial T7SS [38], [39]; however, an orthologous protein does not exist in Firmicutes, perhaps because of the substantial differences in cell envelope architecture between Actinobacteria and Firmicutes. Our data provide evidence that a functionally analogous structure may be formed by the T7SSb system; however, the protein subunits comprising such an assembly remain to be identified. Ultimately, visualization of an intact T7SSb apparatus is required in order to unequivocally demonstrate the existence of a transenvelope complex.

## Experimental Procedures

### Bacterial strains, plasmids and growth conditions

All *S. intermedius* strains used were generated from the sequenced B196 strain [40]. *E. coli* strains XL-1, BL21 Codon Plus and BTH101 were used for plasmid maintenance, protein expression and bacterial two-hybrid assays, respectively. A detailed list of bacterial strains and plasmids used in this study can be found in Table 2, Table 3. *S. intermedius* strains were grown statically in Todd Hewitt broth or on Todd Hewitt agar supplemented with 0.5% yeast extract at 37 °C in the presence of 5% CO_2_. *E. coli* strains used in this study were grown in LB broth at 37 °C in a shaking incubator or on LB agar grown at 37 °C in a static incubator. *S. gallolyticus* ATCC 43143 was grown in Brain Heart Infusion broth at 37 °C in a shaking incubator. *S. intermedius* mutants were generated by replacing the gene to be deleted with a cassette conferring resistance to kanamycin as previously described [10]. Briefly, the antibiotic resistance cassette was cloned between ~ 800 bp of sequence homologous to the regions flanking the gene to be deleted. The DNA fragment containing the cassette and flanking sequences was then linearized by restriction digest, gel purified, and ~ 250 ng of the purified fragment was added to 2 mL of log-phase culture pre-treated for two hours with competence peptide (500 ng/mL) to stimulate natural transformation. Cultures were further grown for 4 h before plating on the appropriate antibiotic. All deletions were confirmed by PCR.

**Table 2:** Strains used in this study

Organism	Genotype	Description	Reference
*S. intermedius* B196	Wild-type		[40]
	ΔSIR_0175::kan^R^	*essC* deletion strain	[10]
	ΔSIR_01486 ΔSIR_01487 ΔSIR_01488::kan^R^	*tipC1*, SIR_1487, *tipC2* deletion strain	This study
	ΔSIR_01486 ΔSIR_01487 ΔSIR_01488 ΔSIR_01489::kan^R^	*telC*, *tipC1*, SIR_1487, *tipC2* deletion strain	This study
*S. gallolyticus* ATCC 43143	Wild-type		[49]
*E. coli* XL-1 Blue	*recA1 endA1 gyrA96 thi-1 hsdR17 supE44 relA1 lac* [F′ *proAB lacI*^q^ Z ∆* M15* Tn*10* (Tet^R^)]	Cloning strain	Agilent
*E. coli* DH5α	F^−^*endA1 glnV44 thi-1 recA1 relA1 gyrA96 deoR nupG* Φ80d*lacZ*ΔM15 Δ(*lacZYA-argF*)U169, *hsdR17*(r_K_^−^ m_K_^+^), λ–	Cloning strain	Novagen
*E. coli* BTH101	F^−^, *cya-99*, *araD139*, *galE15*, *galK16*, *rpsL1* (Str^R^), *hsdR2*, *mcrA1*, *mcrB1*	Bacterial two-hybrid strain	Euromedex
*E. coli* BL21 (DE3) CodonPlus	F^−^*ompT gal dcm lon hsdS*_*B*_(r_B_^−^ m_B_^−^) λ(DE3) pLysS(Cm^R^)	Protein expression strain	Novagen

**Table 3:** Plasmids used in this study.

Plasmid	Relevant features	Reference
pDL277	*Streptococcus-E. coli* shuttle vector, Spec^R^	[50]
pKNT25	B2H expression vector with *plac*, Kan^R^, C-terminal fusion to T25 fragment of CyaA	Euromedex
pUT18C	B2H expression vector with *plac*, Amp^R^, C-terminal fusion to T18 fragment of CyaA	Euromedex
pETDuet-1	Co-expression vector with *lacI*, T7 promoter, N-terminal His_6_ tag in MCS-1, Amp^R^	Novagen
pET29b	Expression vector with *lacI*, T7 promoter, C-terminal His_6_ tag, Kan^R^	Novagen
pDL277::P96_ss-SIR_1489_202-552	*S. intermedius* expression vector for residues 202-552 of TelC fused to a sec signal sequence (ss-TelC_tox_)	[10]
pDL277::P96_ss-SIR1489_202-552_D401A	*S. intermedius* expression vector for ss-TelC_tox_^D401A^	[10]
pDL277::P96_ss-SIR1489_202-552–SIR1488	*S. intermedius* expression vector for ss-TelC_tox_ and TipC1	[10]
pDL277::P96_ss-SIR1489_202-552–SIR1486	*S. intermedius* expression vector for ss-TelC_tox_ and TipC2	This study
pDL277::P96_ss-SIR1489_202-552–SIR1488_F71Q	*S. intermedius* expression vector for ss-TelC_tox_ and TipC1^F71Q^	This study
pDL277::P96_ss-SIR1489_202-552–SIR1488_K93E	*S. intermedius* expression vector for ss-TelC_tox_ and TipC1^K93E^	This study
pDL277::P96_ss-SIR1489_202-552–SIR1488_F71Q_K93E	*S. intermedius* expression vector for ss-TelC_tox_ and TipC1^F71Q, K93E^	This study
pDL277::P96_SIR_1488-V	*S. intermedius* expression vector for TipC1 fused to a C-terminal VSV-G epitope tag	This study
pDL277::P96_SIR_1488_23-204-V	*S. intermedius* expression vector for residues 23-304 of TipC1 (TipC1_ΔTMD_) fused to a C-terminal VSV-G epitope tag	This study
pDL277::P96_SIR_1157-V	*S. intermedius* expression vector for SodA fused to a C-terminal VSV-G epitope tag	This study
pDL277::P96_SIR_1047-V	*S. intermedius* expression vector for LsrS fused to a C-terminal VSV-G epitope tag	This study
pDL277::P96_SIR_1489	*S. intermedius* expression vector for TelC	This study
pDL277::P96_ss-SIR_1489_SIR1488	*S. intermedius* expression vector for ss-TelC and TipC1	This study
pKNT25::sgTelC	B2H expression vector for TelC from *S. gallolyticus*	This study
pUT18C::sgTipC1	B2H expression vector for TipC1 from *S. gallolyticus*	This study
pUT18C::sgTipC2	B2H expression vector for TipC2 from *S. gallolyticus*	This study
pUT18C::sgTipC3	B2H expression vector for TipC3 from *S. gallolyticus*	This study
pUT18C::sgTipC4	B2H expression vector for TipC4 from *S. gallolyticus*	This study
pETDuet-1:: SIR_1489_202-552	*E. coli* expression vector for TelC_tox_	[10]
pET29b::SIR_1488_23-204-V	*E. coli* expression vector for TipC1_ΔTMD_ fused to a C-terminal VSV-G epitope tag (TipC1_ΔTMD_-V)	This study
pET29b::SIR_1486_23-203-V	*E. coli* expression vector for TipC2_ΔTMD_ fused to a C-terminal VSV-G epitope tag	This study
pETDuet-1::SIR_1486_23-203	*E. coli* expression vector for TipC2_ΔTMD_ fused to an N-terminal His_6_-tag	This study
pET29b::SIR_1488_23-204_R56E-V	*E. coli* expression vector for TipC1_ΔTMD_-V R56E variant	This study
pET29b::SIR_1488_23-204_D62R-V	*E. coli* expression vector for TipC1_ΔTMD_-V D62R variant	This study
pET29b::SIR_1488_23-204_F71Q-V	*E. coli* expression vector for TipC1_ΔTMD_-V F71Q variant	This study
pET29b::SIR_1488_23-204_S81Q-V	*E. coli* expression vector for TipC1_ΔTMD_-V S81Q variant	This study
pET29b::SIR_1488_23-204_R87E-V	*E. coli* expression vector for TipC1_ΔTMD_-V R87E variant	This study
pET29b::SIR_1488_23-204_K93E-V	*E. coli* expression vector for TipC1_ΔTMD_-V K93E variant	This study
pET29b::SIR_1488_23-204_R94E-V	*E. coli* expression vector for TipC1_ΔTMD_-V R94E variant	This study
pET29b::SIR_1488_23-204_R96E-V	*E. coli* expression vector for TipC1_ΔTMD_-V R96E variant	This study
pET29b::SIR_1488_23-204_S100Q-V	*E. coli* expression vector for TipC1_ΔTMD_-V S100Q variant	This study
pET29b::SIR_1488_23-204_S112Q-V	*E. coli* expression vector for TipC1_ΔTMD_-V S112Q variant	This study
pET29b::SIR_1488_23-204_S114Q-V	*E. coli* expression vector for TipC1_ΔTMD_-V S114Q variant	This study
pET29b::SIR_1488_23-204_K161E-V	*E. coli* expression vector for TipC1_ΔTMD_-V K161E variant	This study
pET29b::SIR_1488_23-204_K169E-V	*E. coli* expression vector for TipC1_ΔTMD_-V K169E variant	This study
pET29b::SIR_1488_23-204_K186E-V	*E. coli* expression vector for TipC1_ΔTMD_-V K186E variant	This study
pET29b:: SIR_1488_23-203	*E. coli* expression vector for TipC2_ΔTMD_ fused to a C-terminal His_6_-tag	This study
pET29b:: SIR_1488_23-204	*E. coli* expression vector for TipC1_ΔTMD_ fused to a C-terminal His_6_-tag	This study
pET29b:: SIR_1488_23-204_F71Q	*E. coli* expression vector for TipC1_ΔTMD_-his_6_ F71Q variant	This study
pET29b:: SIR_1488_23-204_K93E	*E. coli* expression vector for TipC1_ΔTMD_-his_6_ K93E variant	This study
pET29b:: SIR_1488_23-204_F71Q_K93E	*E. coli* expression vector for TipC1_ΔTMD_-his_6_ F71Q, K93E variant	This study

### DNA manipulation and plasmid construction

*S. intermedius* and *S. gallolyticus* genomic DNA was prepared using a cell lysis buffer containing 20 mg/mL lysozyme (BioShop), 25 mM Tris–HCl (pH 8.0), and 2.5 mM EDTA, and the DNA was purified using the Genomic DNA Mini Kit (Invitrogen). Primers were synthesized and purified by Integrated DNA Technologies (IDT). Q5 polymerase, restriction enzymes and T4 DNA ligase were purchased from New England Biolabs (NEB). Site-specific mutants used in this study were generated by overlap extension PCR. All plasmids were sequenced by Genewiz Incorporated.

### Subcellular fractionation

One liter of each *S. intermedius* strain was grown to an OD_600_ of 0.8 prior to centrifugation at 5524*g* for 15 min. Pelleted cells were then resuspended in lysis buffer containing 25 mM Tris–HCl (pH 8.0), 150 mM NaCl, and 2 mg/mL lysozyme and sonicated (4 × 30-s pulses at 30% amplitude). Insoluble cellular debris was then cleared by centrifugation at 39,191*g* for 30 min, and the resulting supernatant was spun at 200,000*g* for 2 h to isolate the membrane fraction. Aliquots of the supernatant fractions were added to Laemmli loading buffer, whereas the membrane-containing pellet was washed once using 25 mM Tris–HCl (pH 8.0) and 150 mM NaCl buffer prior to dissolving in Laemmli loading buffer. Cytoplasmic and membrane fractions were then subjected to SDS-PAGE and Western blot analysis.

### Protease protection assay

Protease protection assays were performed as recently described for *Streptococcus pneumoniae* with minor modifications [17]. Briefly, 40 mL of the indicated *S. intermedius* strains was grown to OD_600_ = 0.3 prior to harvesting by centrifugation at 4000*g* for 15 min. Cells were washed once in SMM buffer [20 mM maleic acid (pH 6.5), 20 mM MgCl_2_, 0.5 M sucrose] prior to resuspension in 2 mL SMM buffer containing 5 mg/mL lysozyme. Lysozyme digestion was carried out for 20 min at 37 °C followed by washing and resuspension in 1 mL SMM buffer. Aliquots of the resulting spheroplasts were either left untreated, treated with Proteinase K (20 μg/mL), treated with Triton X-100 (1% v/v) or treated with Proteinase K and Triton X-100 for 30 min at room temperature. Proteolysis reactions were quenched using 1 mM PMSF prior to the addition of Laemmli loading buffer. Samples were analyzed by SDS-PAGE and Western blotting.

### Western blotting

Western blot analyses were performed as previously described using rabbit α-VSV-G (Sigma; 1:5000) and rabbit α-TelC (1:3000) [10]. HRP-conjugated goat α-rabbit secondary antibody (Sigma; 1:5000) and ECL substrate (Clarity Max, Bio-Rad) were used for chemiluminescent detection. Western blots were imaged using a ChemiDoc System (Bio-Rad).

### Identification of TipC homologous proteins

To determine TipC1 distribution in bacteria, the amino acid sequence of TipC1 was run through the iterative hidden Markov model search tool JackHMMER against the UniProtKB database. After five iterations, the search converged resulting in the identification of 286 protein sequences. A subset of the gene clusters encoding the identified TipC1 homologous proteins were selected for depiction in Fig. 2.

### Toxicity assays

*S. intermedius* cells were grown to mid-log phase (OD_600_ of 0.6) before competence was induced by the addition of 500 ng of competence-stimulating peptide per milliliter of culture. Cultures were then incubated for 2 h prior to the addition of 1 μg of the indicated plasmids to the media. After an additional 3-h incubation, 100 μL of each culture was plated on selective media.

### Co-purification assays

Fifty milliliters of *E. coli* BL21 cells expressing the indicated plasmids was grown in LB broth to an OD_600_ of 0.6. Protein expression was then induced by adding IPTG to a final concentration of 1 mM following by further incubated for 3 h. Cells were collected by centrifugation at 5524*g* for 10 min and subsequently resuspended in lysis buffer [50 mM Tris–HCl (pH 8.0), 300 mM NaCl, 10 mM imidazole]. Cells were then lysed by sonication, and cellular debris was removed by centrifugation at 39,191 *g* for 30 min. Aliquots of the cleared lysate were added to Laemmli loading buffer for downstream Western blot analysis of the input fraction. One hundred microliters of Ni-NTA slurry (Qiagen) was then added to the remaining cell lysate and incubated at room temperature for 1 h. The beads were then washed three times with 10 mL of wash buffer [20 mM Tris–HCl (pH 8.0), 300 mM NaCl, 10 mM imidazole] by iterative rounds of centrifugation at 700*g* for 2 min followed by removal of the supernatant. Proteins bound to the Ni-NTA resin were then eluted by adding 500uL of elution buffer (20 mM Tris–HCl, 150 mM NaCl, 400 mM imidazole) followed by a final spin at 700*g* to remove the resin. The eluate was then added to Laemmli sample buffer and was analyzed, along with the input fractions, by Western blot.

### Bacterial two-hybrid analyses

*E. coli* BTH101 cells were co-transformed with plasmids encoding the T25 and T18 fragments of *Bordetella pertussis* adenylate cyclase fused to *Sg*TelC and *Sg*TipC1–4, respectively. Stationary phase cells were then plated on LB agar containing 40 μg/mL X-gal, 0.5 mM IPTG, 50 μg/mL kanamycin and 150 μg/mL carbenicillin and grown for 30 h at 30 °C. Plates were imaged using an iPhone 7 (Apple Inc.). A representative image of each two-hybrid experiment is shown. Three independent replicate experiments were performed for each pairwise combination and yielded comparable results.

### Protein expression and purification

Two liters of *E. coli* BL21 CodonPlus cells expressing pETDuet-1::*tipC2*_*ΔTMD*_ were grown at 37 °C in 2xYT broth an OD_600_ of 0.6 prior to induction of protein expression with 1 mM IPTG. Following further incubation at 37 °C for 4 h, cells were harvested by centrifugation and flash frozen. Frozen cells were thawed using lysis buffer [50 mM Tris–HCl (pH 8.0), 300 mM NaCl, 10 mM imidazole] and lysed by sonication (6 × 30 s pulses at 30% amplitude). Insoluble cellular debris was then cleared by centrifugation and the TipC2-containing supernatant was applied to a 5 mL HisTrap™ FF Ni-NTA cartridge connected to an AKTA FPLC purification system (GE Healthcare). Unbound proteins were removed by extensive washing of the column in lysis buffer, and TipC2_ΔTMD_ was eluted using a linear imidazole gradient to a final concentration of 400 mM. Ni-NTA purified fractions of TipC2_ΔTMD_ were pooled, and the protein was further purified using a 16/600 HiLoad S200 size exclusion column (GE Healthcare) run in 20 mM Tris–HCl (pH 8.0) and150 mM NaCl. Selenomethionine-incorporated TipC2_ΔTMD_ was expressed an purified in an identical manner except that cells were grown in SelenoMethionine Medium Complete (Molecular Dimensions), and all purification buffers contained 1 mM tris(2-carboxyethyl)phosphine.

### Crystallization and structural analyses

Size exclusion-purified TipC2_ΔTMD_ was concentrated to 25 mg/mL by spin filtration prior to crystallization (10 kDa MWCO; Millipore). TipC2_ΔTMD_ at a concentration of 25 mg/mL was screened against commercially available sparse matrix crystallization kits (MCSG1–4; Anatrace). After several days of incubation at room temperature, crystals of TipC2_ΔTMD_ grew in 100 mM Tris–HCl (pH 8.5) and 25% w/v polyethylene glycol 3350. Optimization of native TipC2_ΔTMD_ was not pursued because selenomethionine-incorporated TipC2_ΔTMD_ also readily crystallized in this condition. Single crystals of selenomethionine incorporated TipC2_ΔTMD_ were obtained by the streak seeding method, and following cryoprotection of single crystals in the crystallization buffer supplemented with 20% ethylene glycol, a 1.8-Å data set was collected at beamline 5.0.2 at the Advanced Light Source (360 images, 1.0° Δ φ oscillation, 1.0-s exposure and 250-mm crystal-to-detector distance). X-ray diffraction data were merged, integrated and scaled using the *xia2* system [41].

X-ray phases were obtained by the selenium SAD technique using the AutoSol wizard built into the Phenix GUI [42]. The resulting electron density map was of sufficient quality to allow for automated model building of the complete structure using Phenix AutoBuild [43]. Minor model adjustments were made manually in Coot between iterative rounds of refinement using Phenix.refine [44], [45]. The final model was refined to an *R*_work_ of 17.0% and an *R*_free_ of 19.4%.

### Homology modeling

A homology model of the TipC1_ΔTMD_ was obtained using the structure prediction server I-Tasser using the TipC2_ΔTMD_ structure as a template. The I-Tasser-generated model of TipC1_ΔTMD_ had sequence coverage of 99% and a normalized *Z*-score of 10.0 [23].

### Circular dichroism spectroscopy

Circular dichroism spectra were acquired using an AVIV model 4010 circular dichroism spectrometer (AVIV Associates, Lakewood, NJ). Prior to data acquisition, protein samples were buffer exchanged into 2 mM Hepes and 15 mM NaCl. Samples were then transferred to a quartz cell with a 1-mm path length, and data were collected at 25 °C. For each protein sample, spectra were averaged from three scans.

### Lipid II phosphatase assay

The digestion of Lys-type lipid II (gift from Eefjan Breukink, University of Utrecht) was assessed by thin-layer chromatography as previously described [46]. Briefly, TelC_tox_ alone or TelC_tox_ with 1.2 molar equivalents of TipC1_ΔTMD_, TipC1_ΔTMD_^F71Q^, TipC1_ΔTMD_^K93E^ or TipC1_ΔTMD_^F71Q, K93E^ was incubated in a total volume of 50 μl with 2 nmol lipid II in 150 mM KCl, 0.1% Triton X-100 and 2 mM CaCl_2_ for 90 min at 37 °C. Lipids were extracted with *n*-butanol/pyridine acetate (2:1) at pH 4.2 and resolved on an HPTLC silica gel 60 plate (Millipore) developed with chloroform/methanol/ammonia/water (88:48:1:10). Compounds were stained with iodine, and bands were quantified by the ImageJ software.

### Growth curves

For *S. intermedius* growth curves, overnight cultures of the indicated strains were sub-inoculated into THYB to a starting OD_600_ of 0.01. Cultures were grown statically at 37 °C in the presence of 5% CO_2_, with OD_600_ measurements being taken at the indicated time points.

### Secretion assay

*S. intermedius* strains were grown to an OD_600_ of 0.7 prior to harvesting by centrifugation at 10,000*g* for 10 min. Cell and supernatant fractions were prepared as described previously and analyzed by Western blot analysis [10].

### Protein Data Bank accession numbers

The atomic coordinates and structure factors (code 6DHX) have been deposited in the Protein Data Bank (http://wwpdb.org/).

## Appendix A Supplementary data

Supplementary figures Image 1
