# Supplementary material for: Molecular Basis for Immunity Protein Recognition of a Type VII Secretion System Exported Antibacterial Toxin
Source: J Mol Biol. 2018 Oct 19;430(21):4344–58. doi: 10.1016/j.jmb.2018.08.027 (PMC6193138; doi:10.1016/j.jmb.2018.08.027)
Supplement: Supplementary file 1 — Supplementary figures [file mmc1.docx]

**Supplementary Data**

**Molecular basis for immunity protein recognition of a type VII secretion system exported antibacterial toxin**

Timothy A. Klein^1,2^, Manuel Pazos^3^, Michael G. Surette^1,2,4^, Waldemar Vollmer^3^ and John C. Whitney^1,2*^

^1^Michael DeGroote Institute for Infectious Disease Research, McMaster University, Hamilton, Canada, L8S 4K1

^2^Department of Biochemistry and Biomedical Sciences, McMaster University, Hamilton, Canada, L8S 4L8

^3^Centre for Bacterial Cell Biology, Institute for Cell and Molecular Biosciences, Newcastle University, Newcastle upon Tyne, United Kingdom, NE2 4HH

^4^Department of Medicine, Farncombe Family Digestive Health Research Institute, McMaster University, Hamilton, Canada, L8S 4K1


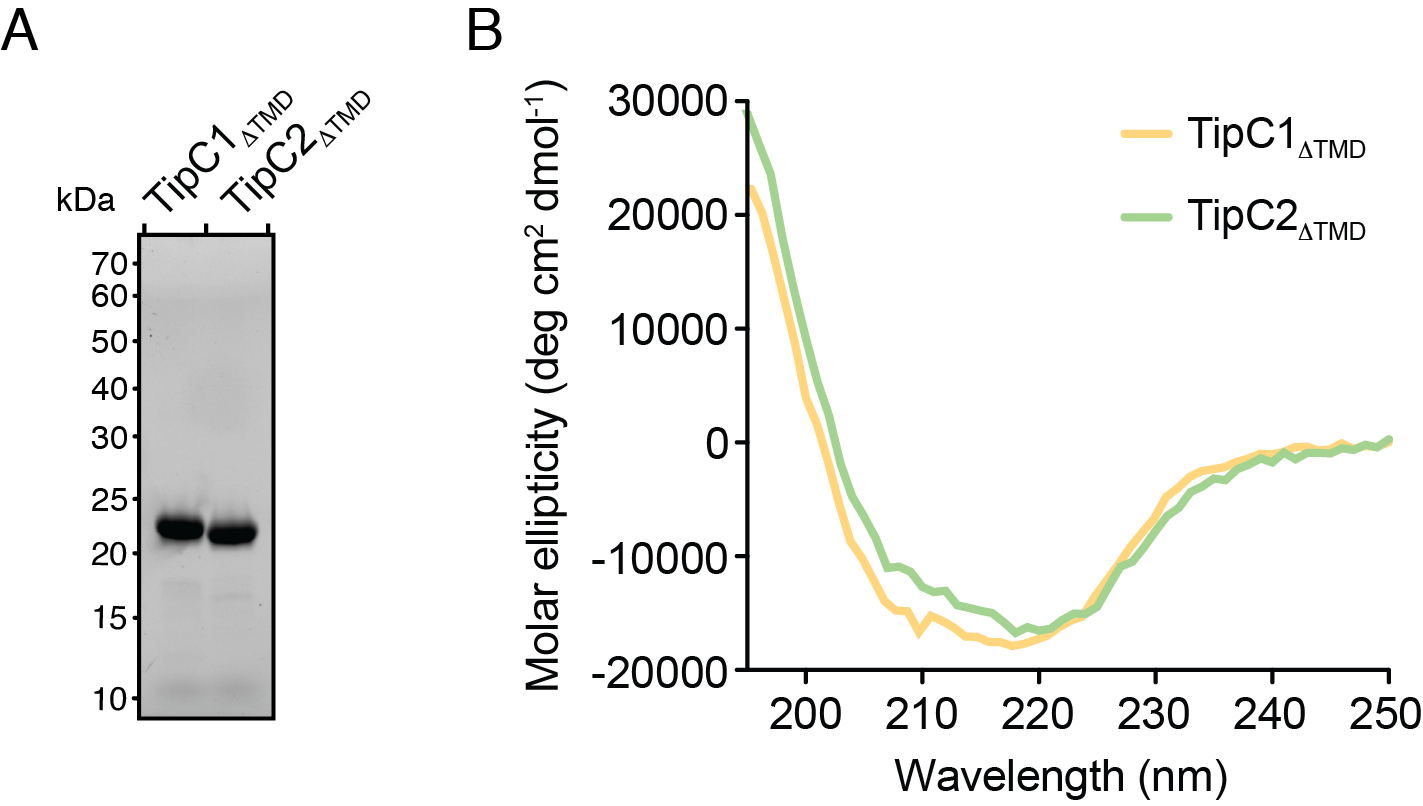


**Figure S1. TipC1_ΔTMD_ and TipC2_ΔTMD_ are comprised of highly similar secondary structure elements.** (A) SDS-PAGE analysis of purified TipC1_ΔTMD_ and TipC2_ΔTMD_ used for circular dichroism analysis. (B) Far-UV circular dichroism spectra of TipC1_ΔTMD_ and TipC2_ΔTMD_.


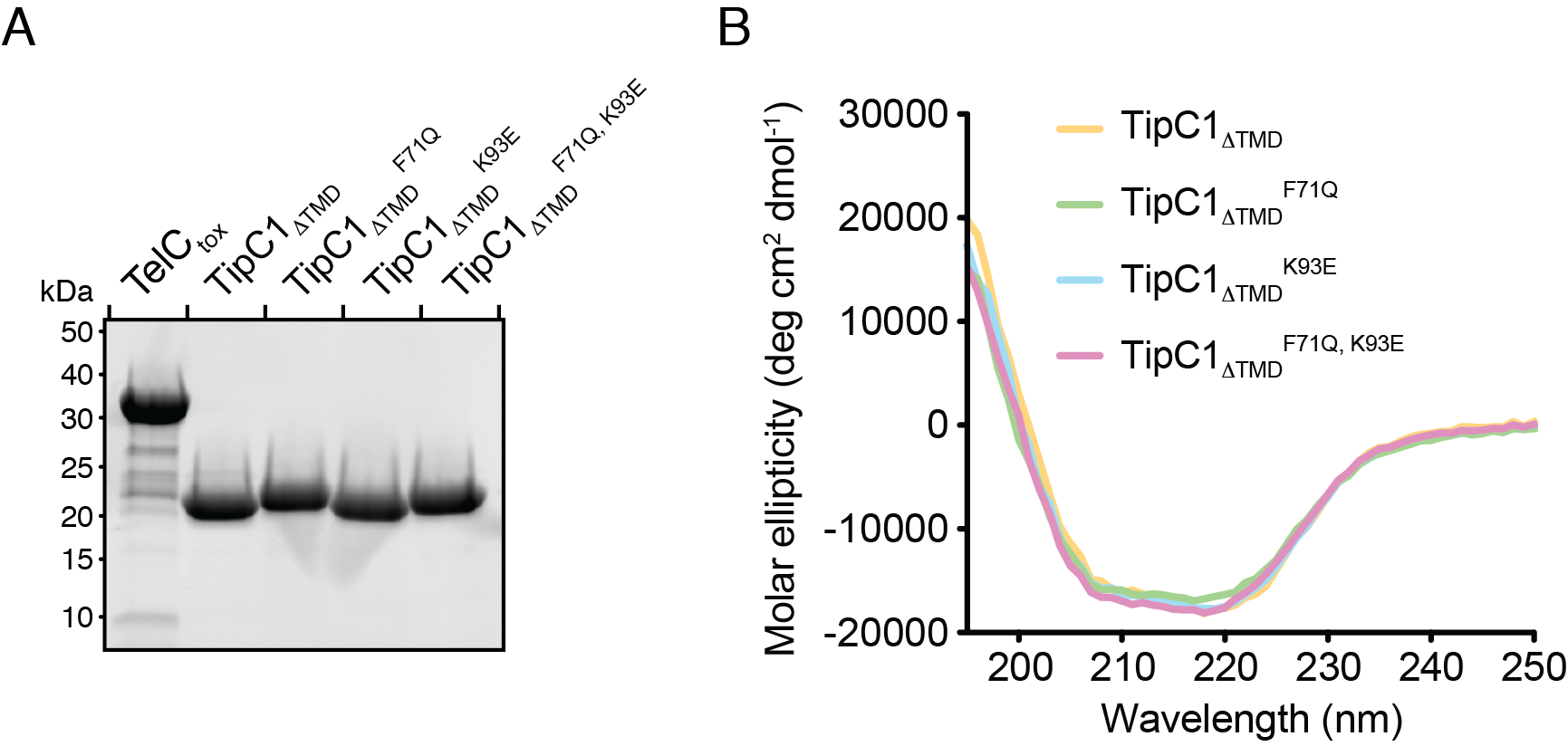


**Figure S2. TipC1_ΔTMD_ and the indicated TipC1_ΔTMD_ site-specific variants are comprised of highly similar secondary structure elements.** (A) SDS-PAGE analysis of purified TelC_tox_, TipC1_ΔTMD_ and the indicated TipC1_ΔTMD_ site-specific variants used for lipid II phosphatase assays. (B) Far-UV circular dichroism spectra of TipC1_ΔTMD_ and the indicated TipC1_ΔTMD_ site-specific variants.
